# Neurocysticercosis among patients with first time seizure in Northern Namibia

**DOI:** 10.11604/pamj.2016.24.127.8908

**Published:** 2016-06-09

**Authors:** Innocent Lule Segamwenge, Ngalyuka Paul Kioko, Celia Mukulu, Ogunsina Jacob, Wanzira Humphrey, Josephine Augustinus

**Affiliations:** 1 Department of Internal Medicine, Intermediate Hospital Oshakati, Oshakati, Namibia; 2 Department of Radiology, Intermediate Hospital Oshakati, Oshakati, Namibia; 3 Infectious Disease Collaboration, Makerere University

**Keywords:** Neurocysticercosis, first-time seizure, epilepsy

## Abstract

**Introduction:**

Neurocysticercosis is a common cause of seizures in low resource countries. There is a paucity of data regarding the extent of this infection in Namibia. There are multiple causes of *First-time seizure* including electrolyte abnormalities, infections, trauma, drugs, alcohol and many times no apparent cause can be found. We sought to describe the burden of Neurocysticercosis among individuals with a first-time seizure in Namibia.

**Methods:**

We recruited 221 patients with a First-time seizure who presented to the Intermediate Hospital Oshakati between August 2012 and March 2014. Patients with seizures due to identifiable causes like trauma, electrolytes, intoxications and meningitis were excluded. Brain CT scans were done, blood serological testing of Neurocysticercosis antibodies, Physical examination and demographic variables were collected. Data was entered into Epidata version 3.1 and transferred to stata version for analysis.

**Results:**

Ninety-six (96) of the participants had evidence of Neurocysticercosis on Brain CT scan representing a prevalence of 51.41%. Consumption of pork and rearing of pigs in the homestead were significant factors associated with Neurocysticercosis in our study population with odds of 3.48(1.45-8.33) and 2.07(1.11-3.86) respectively. Serological testing for Cyticercosis IgG had a sensitivity of 65.93% and Specificity of 96.51%. The positive and negative predictive values were 95.2% and 72.81% respectively.

**Conclusion:**

Neurocysticercosis is a common cause of Index seizures in Northern Namibia, living in a rural area, rearing pigs in homesteads, eating pork and poor sanitary practices are the major risk factors for this illness.

## Introduction

Neurocystsicercosis (NCC) is a neurological disorder resulting from infection of the brain with larval forms of Taenia Solium [1]. NCC is a major problem in many developing countries where the infection is considered to be endemic [2–4]. In these countries NCC contributes significantly to the burden of epilepsy [5]. The infection is most prevalent in communities that feed on pork, where sanitary conditions are poor, and pigs are allowed to roam freely in the vicinity of people's habitats [5]. An individual presenting with an unprovoked seizure is said to be having a new onset seizure. In high-income countries the utility of Brain imaging for individuals presenting with new onset seizures has been highly debated. In these settings the diagnostic yield of Brain computed Tomography scan imaging was found to be only useful in patients who had an abnormal neurological examination, elderly and those with abnormal electroencephalograms [6, 7]. Similar data from low-income countries with high burden of infectious causes of seizures is not available. This study was conducted to provide data on the prevalence of Neurocystsicercosis among patients with new onset seizures and also to identify associated risk factors in Northern Namibia.

## Methods

This was an analytical cross sectional study conducted among patients with a *first time Seizure* presenting to the casualty and Medical in-patient wards of the Intermediate Hospital Oshakati Hospital, a referral hospital in Namibia, between August 2012 and March 2014. We recruited 221 patients presenting with generalized seizures without a prior history of Epilepsy, who were 12 years and above and had provided informed consent. We excluded Patients with seizure disorders confirmed to be secondary to confirmed brain pathology Meningitis, brain trauma, tumours, cerebrovascular accident or birth asphyxia and those who were below 12 years of age. This was done through seeking for specific history on alcohol use, recent brain injury as well physical examination for signs of meningitis and any neurological deficits. All patients underwent a physical examination; a questionnaire was administered to collect demographic data and selected risk factors like area of residence, rearing of pigs, consumption of pork and sanitary practices. Blood was collected to test for Urea, creatinine and electrolytes as well as cyticercosis serology using an Elisa test. A brain computed tomogram was also performed. Data on clinical and demographic variables was entered in Epidata version 3.1 and was later transferred to stata version 13 for analysis. The prevalence of NCC with accompanying 95% confidence interval was estimated by taking the proportion of participants who were diagnosed with NCC by Brain CT scan among all participants with new seizure episode. Our gold standard for the diagnosis of Neurocysticercosis (NCC) was the presence of lesions highly suggestive of NCC on computed tomography scan (CT scan) [8]. A forward fitting multivariate logistic regression model was used to estimate the odds ratio with 95% CI to assess the contribution of selected factors to NCC. In all analysis, a p-value of = 0.05 was taken as statistically significant. The research and Ethics committee of the Ministry of Health and Social services of Namibia provided approval for the study.

## Results

**Figure 1:**
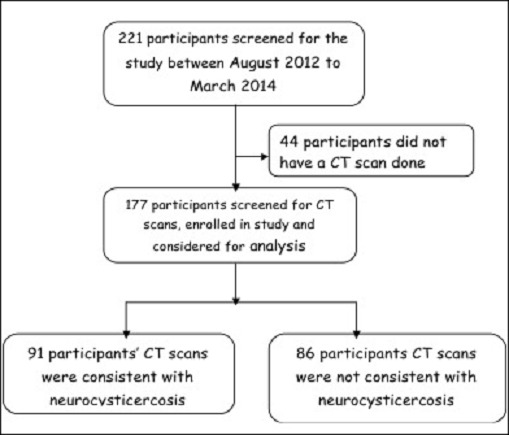
Shown is the flow diagram of the enrolment of study participants

We screened two hundred and twenty one (221) patients who presented to the casualty and Medical in-patient department of the intermediate hospital Oshakati. Forty-four (44) patients who did not have a Brain CT scan done were excluded from the final analysis. One hundred seventy seven (177) were included in our study (Figure 1). The majority of the participants 133(74%) were between the ages of 12 to 30 years with a mean age of 31.47(±17.14), were residing in rural areas 149 (84%), ate pork 145 (82%) and were rearing pigs in their homesteads 112 (63%). The use of the countryside for sanitary purposes was found in 96 (54%) of the participants (Table 1).

**Table 1:** Shown are the baseline characteristics of study participants and selected risk factors predisposing patients to Neurocysticercosis. Most patients were below 30 years of age and living in rural areas with poor sanitary facilities and reared pigs in the homesteads and also ate pork

Variable	Participant distribution	
	Frequency	Percentage
**Age categories (years)**		
12-15	16	9.03
16-30	115	64.97
31-60	31	17.51
> 60	15	8.47
**Sex**		
Male	87	48.86
Female	90	51.14
Living area		
Urban	28	15.82
Rural	149	84.18
**Sanitation**		
House toilets	17	9.60
Outside latrines	60	33.90
In country side	96	54.24
Unknown	4	2.26
Piggery		
Yes	112	63.28
No	65	36.72
**Eat pork**		
Yes	145	81.92
No	30	16.95
Unknown	2	1.13

### Prevalence of NCC

Ninety-six (96) of the participants had evidence of Neurocysticercosis on Brain CT scan representing a prevalence of 51.41%.

### Factors associated with neurocysticercosis among patients with new onset seizures

Consumption of pork and rearing of pigs in the homestead were significant factors associated with Neurocysticercosis in our study population with odds of 3.48(1.45-8.33) and 2.07(1.11-3.86) respectively. The use of the countryside as toilet facilities was also a significant risk factor associated with Neurocysticercosis with OR of 2.92(1.00-8.58) (Table 2).

**Table 2:** Shown in the table are selected risk factors associated Neurocysticercosis among patients with first-time seizures. Consumption of pork, rearing of pigs in the homestead and use of the countryside, as toilet facilitieswere significant factors associated with Neurocysticercosis in our study population

Variable	Presence of Neurocysticercosis				
	Yes	No	Crude OR (95% CI)	Adjusted OR(95%CI)+	p-value
**Age categories (years)**					
≥ 15	8(61.54)	5(38.46)	Reference	Reference	0.295
16-30	57(49.57)	58(50.43)	0.61(0.19-1.99)	2.51(0.45-14.06)	0.629
31-60	15(48.39)	16(51.43)	0.59(0.16-2.20)	1.62(0.23-11.31)	0.312
> 60	10(66.67)	5(33.33)	1.25(0.27-5.89)	3.31(0.32-33.78)	
**Sex**					
Male	40(46.51)	46(53.49)			
Female	51(56.67)	39(43.33)	1.50(0.83-2.73)	0.90(0.38-2.13)	0.802
**Living area**					
Urban	16(57.14)	12(42.86)			
Rural	75(50.34)	74(49.66)	0.76(0.34-1.72)	0.35(0.10-1.24)	0.105
**Sanitation**					
House toilets	6(35.29)	11(64.71)	Reference	Reference	
Outside latrines	23(38.33)	37(61.67)	1.14(0.37-3.50)	0.75(0.17-3.31)	0.705
In country side	59(61.46)	37(38.54)	2.92(1.00-8.58)	2.16(0.51-9.12)	0.294
**Piggery**					
No	26(40.00)	39(60.00)			
Yes	65(58.04)	47(41.96)	2.07(1.11-3.86)	1.85(0.68-5.00)	0.225
**Eat pork**					
No	8(26.67)	22(73.33)			
Yes	81(55.86)	64(44.14)	3.48(1.45-8.33)	3.23(0.81-12.97)	0.098

### Performance of serological testing for neurocysticercosis

**Figure 2:**
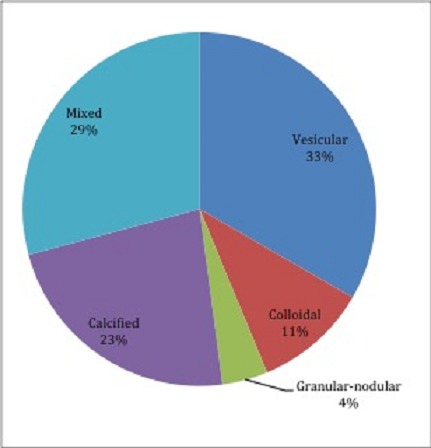
Shown is the distribution of neurocysticercosis lesions based on brain CT appearance and description. The most common brain lesions were vesicular cysts and the mixed type

Serological testing for cyticercosis IgG had a sensitivity of 65.93% and Specificity of 96.51%. The positive and negative predictive values were 95.2% and 72.81% respectively (Table 3). Among patients with confirmed Neurocysticercosis on Brain CT scan, 33% and 29% had Vesicular and Mixed lesions respectively (Figure 2).

**Table 3:** Shown in the table is the sensitivity, specificity as well positive and negative predictive values comparing the performance of serology with Brain CT as the gold standard in diagnosis neurocysticercosis

Test	Neurocysticercosis by CT Scan					
	Yes	No	Sensitivity	Specificity	Positive Predictive Value	Negative Predictive Value
Serological tests						
Positive	60	3				
Negative	31	83	65.93	96.51	95.24	72.81

## Discussion

In developing countries the causes of seizures are often preventable. The major causes of seizures in Africa are childhood febrile convulsions and various infections involving the central nervous system [9, 10]. Neurocysticercosis has been frequently identified as one of the infections frequently associated with epilepsy [11–14]. In our study the prevalence of Neurocysticercosis among patients with a new onset seizure was 51.41%. Most of the patients in our study had evidence of active NCC in the form of Vesicular lesions 33%, colloidal 11% and granular-nodular 4%. Our findings are similar to a study conducted in South Africa, which found a prevalence of 61.1% [15]. Another neuroimaging study conducted in rural Tanzania found evidence of Neurocysticercosis among 17.9% of patients with Epilepsy [16]. The findings in these epilepsy studies may not be directly comparable to our study, which instead looked at patients with New-onset seizures. However, what is clear from our findings is that Neurocysticercosis is a very common cause of seizures in most Sub-Saharan African countries. It appears that performing a brain CT scan on everyone with a new-onset seizure would be a cost effective intervention in this setting. This is in contrast to findings in high-income countries where a strategy of Brain CT imaging in the evaluation of new-onset seizures has been has been debated [6, 7]. However, Brain CT imaging is not always available for most patients with seizures in low-income countries. In our study patients with Neurocysticercosis were more likely to be between the ages 15 and 30 years, residing in a rural area, rear pigs in the homestead and associated poor sanitary practices. A study conducted in Togo found high rates of NCC in a community, which fed on pork and allowed free roaming of pigs in vicinity of people's habitations [17]. Knowledge of such a high prevalence of NCC and these risk factors provides an opportunity for public health intervention in these affected communities. The sensitivity of serological testing for cyticercosis was 66% with a specificity of 97% and a positive predictive value of 95%. This high sensitivity may be explained by large number of patients with active lesions in our study. Investigators in South Africa have described differences in sensitivity between active and calcified lesions [15]. In Sub-Saharan Africa, especially in rural settings, most patients may not have access to Brain CT imaging. In our Namibian setting this provides an opportunity for screening patients with seizures who may not have access to Brain CT scan. Those with positive serology may be given anthelminthic and Brain Imaging performed as it becomes available.

## Conclusion

Neurocysticercosis is a common cause of New-onset seizures in Northern Namibia, living in a rural area, rearing pigs in homesteads, eating pork and poor sanitary practices are the major risk factors for this illness. In Namibia there is a role of using serological testing cysticercosis among patients with New-onset seizures especially where Brain Imaging may be not be accessible.

### What is known about this topic

- Epilepsy and seizures in Africa are largely due to preventable causes like CNS infections and poor maternal- child services leading to birth asphyxia;
- Neurocysticercosis is a common cause of epilepsy in low resource countries in Sub-Saharan Africa and Latin America;
- For most African countries like Namibia there is no local data on the disease and hence no routine screening or community interventions place to address this problem.

### What this study adds

- The study adds to the pool of knowledge available that Neurocysticercosis largely remains a neglected disease even in a Middle-income country like Namibia;
- The link between Neurocysticercosis and Epilepsy is very clear; however there is no data on the Prevalence of this condition among patients with new-onset seizures;
- The study hence calls for Brain CT imaging in most patients presenting with seizures for the first time in our Namibian setting and perhaps in most endemic areas.
